# Consensus Defined Diastolic Dysfunction and Cardiac Postoperative Morbidity Score: A Prospective Observational Study

**DOI:** 10.3390/jcm10215198

**Published:** 2021-11-07

**Authors:** Bonnie Kyle, Mateusz Zawadka, Hilary Shanahan, Jackie Cooper, Andrew Rogers, Ashraf Hamarneh, Vivek Sivaraman, Sibtain Anwar, Andrew Smith

**Affiliations:** 1 Perioperative Medicine, Barts Heart Centre, St. Bartholomew’s Hospital, London EC1A 7BE, UK; bonnie.kyle@nhs.net (B.K.); dr.amrogers@gmail.com (A.R.); a.hamarneh@ucl.ac.uk (A.H.); s.anwar@qmul.ac.uk (S.A.); andrew.smith84@nhs.net (A.S.); 2 NIHR Biomedical Research Centre, William Harvey Research Institute, Barts, Queen Mary University of London, London E1 4NS, UK; jackie.cooper@qmul.ac.uk; 3 2^nd^ Department of Anesthesiology and Intensive Care, Medical University of Warsaw, 02-091 Warsaw, Poland; 4 Polish National Agency for Academic Exchange, 00-635 Warsaw, Poland; 5 Department of Anaesthesia and Critical Care, Papworth Hospital NHS Foundation Trust, Papworth Everard, Cambridge CB2 0AY, UK; hshanahan@nhs.net; 6 Outcomes Research Consortium, Cleveland Clinic, Cleveland, OH 44195, USA

**Keywords:** diastolic dysfunction, echocardiography, transesophageal, morbidity, cardiac surgery

## Abstract

Diastolic dysfunction is associated with major adverse outcomes following cardiac surgery. We hypothesized that multisystem endpoints of morbidity would be higher in patients with diastolic dysfunction. A total of 142 patients undergoing cardiac surgical procedures with cardiopulmonary bypass were included in the study. Intraoperative assessments of diastolic function according to the 2016 American Society of Echocardiography and European Association of Cardiovascular Imaging guidelines using transesophageal echocardiography were performed. Cardiac Postoperative Morbidity Score (CPOMS) on days 3, 5, 8, and 15; length of stay in ICU and hospital; duration of intubation; incidence of new atrial fibrillation; 30-day major adverse cardiac and cerebrovascular events were recorded. Diastolic function was determinable in 96.7% of the dataset pre and poststernotomy assessment (*n* = 240). Diastolic dysfunction was present in 70.9% (*n* = 88) of measurements before sternotomy and 75% (*n* = 93) after sternal closure. Diastolic dysfunction at either stage was associated with greater CPOMS on D5 (*p* = 0.009) and D8 (*p* = 0.009), with CPOMS scores 1.24 (*p* = 0.01) higher than in patients with normal function. Diastolic dysfunction was also associated with longer durations of intubation (*p* = 0.001), ICU length of stay (*p* = 0.019), and new postoperative atrial fibrillation (*p* = 0.016, OR (95% CI) = 4.50 (1.22–25.17)). We were able to apply the updated ASE/EACVI guidelines and grade diastolic dysfunction in the majority of patients. Any grade of diastolic dysfunction was associated with greater all-cause morbidity, compared with patients with normal diastolic function.

## 1. Introduction

Diastolic dysfunction refers to the inability of the ventricle to relax to its original unstressed state [1]. It is present in up to 50% of elderly patients [2,3] in the setting of cardiac surgery. Diastolic dysfunction is associated with major adverse cardiovascular events [4,5], respiratory complications, and length of stay after cardiac surgery and in-hospital mortality [6,7] and is an independent predictor of major adverse outcomes after vascular [8] and noncardiac surgery [9,10].

Analysis of outcomes following cardiac surgery tends to focus on endpoints of immediate morbidity (inotropic requirements, difficulty weaning from cardiopulmonary bypass [11,12]), major morbidity (stroke, renal failure, reoperation, hospital length of stay), and mortality [4,5,13,14]. As mortality from cardiac surgery continues to fall, however, understanding and quantifying wider measurements of morbidity assume greater importance. The contribution of diastolic dysfunction to morbidity endpoints beyond “major adverse” following cardiac surgery is less well defined, and yet likely to impact the length of stay.

The Cardiac Postoperative Morbidity Score (CPOMS) [15] is a validated tool for numerically quantifying total morbidity burden on days 3, 5, 8, and 15 after cardiac surgery. This multisystem tool assesses and scores morbidity under 13 clinical domains of pulmonary, infectious, renal, gastrointestinal, cardiovascular, neurological, hematological, wound, pain, electrolyte, endocrine, assisted ambulation, and review (Appendix A).

In this prospective observational study, we hypothesize that morbidity is greater for patients with diastolic dysfunction, as defined by recent consensus-based international guidelines [16] when compared with those with normal diastolic function. The morbidity measures reported are CPOMS (on days 3, 5, 8, and 15, as per the validation for this tool), length of stay in intensive care and hospital, duration of intubation, the incidence of new postoperative atrial fibrillation, and major adverse cardiac and cerebrovascular events (MACCE).

## 2. Materials and Methods

### 2.1. Patients

**Figure 1:**
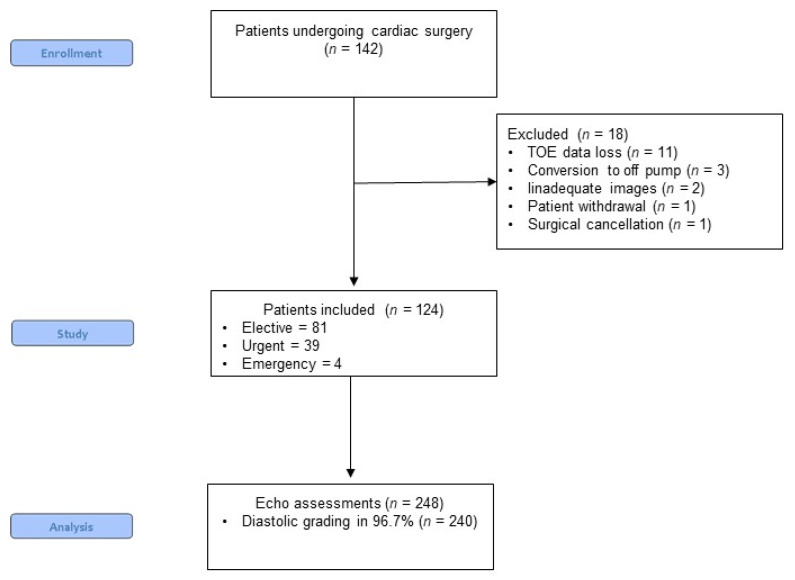
CONSORT diagram of patient recruitment.

In total, 142 patients undergoing cardiac surgery from November 2014 to December 2016 provided consent for participation in this study, and a total of 124 patients were included in the final analysis (in 11 patients, the echocardiography dataset was not fully available due to technical issues, 3 patients were converted to the off-pump procedure, in 2 patients, there was not adequate imaging, and a single patient withdrew their consent and there was one surgical cancelation surgery) (Figure 1).

The study population consisted of adult patients undergoing elective, urgent (surgery within the current admission) or emergency (operation before the beginning of the next working day) at high-volume UK cardiac center (Appendix B). A range of operative urgency and operative procedures were recruited to represent a typical cardiac surgical population, and to assess the applicability of guidelines to assess diastolic function to a perioperative population routinely examined with transesophageal echocardiography (TEE). Surgery included isolated coronary artery bypass grafting (CABG), isolated valve, or CABG plus valve procedures with cardiopulmonary bypass.

### 2.2. Intraoperative Management

Patients were not premedicated. The induction of anesthesia was standardized according to the local protocol. Mean arterial pressure was maintained between 50 and 80 mmHg using metaraminol or phenylephrine. A TEE probe was introduced after induction of anesthesia. Choice of inotropic support (including the use of phosphodiesterase inhibitors), red cell and product transfusion, volume administration, pacing, and management of separation from bypass were at the discretion of the operating team.

### 2.3. Echocardiography

Three cardiovascular ultrasound machines using a multiplane transesophageal echo probe were available, including a Phillips iE33 (Philips Medical Systems, Andover, MA, USA), a Siemens Acuson CV70 (Siemens Medical Solutions, Munich, Germany), and a GE Vivid E9 (GE Medical Systems, Boston, MA, USA). All echocardiographic data were collected, analyzed, and interpreted by one of three EACVI-accredited TEE operators blinded to the patients’ data. Comprehensive datasets were acquired for each patient at two stages intraoperatively: before sternotomy, and after sternal closure (i.e., when the chest was closed). Measurements were taken during hemodynamic stability and without the need for manipulation of fluids or vasoactive drugs. Sweep speeds were set at 50–100 mm/s, and measurements were recorded during apnea. To ensure high-quality data collection, all variables were measured during apnea, the average of three cardiac cycles was used for analysis, and cardiac cycles with extrasystoles on electrocardiogram were excluded. Where apnea was clinically inappropriate, several measurements were performed, and averaged values were calculated (Appendix C).

### 2.4. Assessment of Diastolic Dysfunction

Left ventricular diastolic function was assessed according to the 2016 American Society of Echocardiography (ASE) and European Association of Cardiovascular Imaging (EACVI) guidelines for the evaluation of Left Ventricular Diastolic Dysfunction [16]. The following outcomes of diastolic function assessment were possible: normal diastolic function (DFN), grade 1 diastolic dysfunction (DD1), grade 2 diastolic dysfunction (DD2), grade 3 diastolic dysfunction (DD3), diastolic dysfunction with raised left atrial pressure (DDRLAP), diastolic dysfunction of indeterminate grade (DDIDG), indeterminate if diastolic dysfunction present (IDDD). (Process of evaluating diastolic function is shown in Appendix C).

### 2.5. Data Collection

Data relating to patient characteristics, comorbidities, medications, intraoperative transesophageal echo findings, and postoperative outcomes were prospectively collected on case report forms and entered onto REDCap, a secure database. Prolonged length of stay was defined as 11 or more days, as described in a large UK audit of the length of stay after cardiac surgery [17].

### 2.6. Statistical Analysis

Initially, the primary outcome was a change in diastolic function following the use of enoximone. During this study—following recruitment of only 80 patients—our two institutions merged, leading to a change in pharmacy and formularies, replacing enoximone entirely (with milrinone.) As a result, we were unable to continue assessing the impact of enoximone and instead report the effect of diastolic dysfunction, since defined by ASE/ECAVI, on morbidity with a primary outcome of CPOMS at days 3, 5, 8, and 15. Secondary outcomes remain unchanged: length of stay in intensive care and hospital, duration of intubation, the incidence of new postoperative atrial fibrillation, and major adverse cardiac and cerebrovascular events.

Power calculations were made using published CPOMS data, where each unit increase in CPOMS summary score was associated with a subsequent increase in length of stay. To detect a one-unit increase in mean (SD) CPOMS, required sample sizes were D3 *n* = 42, D5 *n* = 121, D8 *n* = 37, and D15 *n* = 57 (alpha 0.05, beta 0.2, power 0.8), requiring a sample size of 121, plus an additional 20 allowing for data attrition.

Descriptive statistics were applied to examine outcomes of interest. Lavene’s and the Shapiro–Wilk tests were performed to evaluate the data distribution. Normally distributed data were presented as mean ± standard deviation, whereas nonparametric data were presented as median and interquartile ranges. For continuous variables where parameters were not normally distributed, Mann–Whitney or Kruskal–Wallis test was used. For categorical variables, Fisher’s exact test was used. To examine the severity of diastolic dysfunction on composite morbidity outcomes, trends across the three groups (normal diastolic function, diastolic dysfunction with normal left atrial pressure, and diastolic dysfunction with raised left atrial pressure) were assessed using a nonparametric test for trend for continuous data, and an exact logistic regression model for categorical data.

To determine if the presence of diastolic dysfunction was independently associated with hospital length of stay, an all-subset regression was used to select the best model from the available covariates. Hospital length of stay was log-transformed and observed differences converted to percentages. All variables in Table 1 (except for RWMAs and EF where numbers were too small) were considered for inclusion in the model, with the final subset of variables selected using the Akaike information criterion with correction for small samples (AIC). Diastolic dysfunction was then added to the model to obtain the percentage change in length of stay after adjustment for the selected covariates. A multiple logistic regression model was also fitted using the selected covariates with the length of stay above/below 11 days. For each outcome, a two-sided *p* value less than 0.05 was considered significant. Statistical analyses were performed using Stata Version 14 (StataCorp LLC, College Station, TX, United States) software.

## 3. Results

Patients’ baseline characteristics are shown in Table 1. Patients with any grade diastolic dysfunction had significantly more comorbidities. There was a high incidence of hyperlipidemia, hypertension, and systolic heart failure.

From a total of 248 assessments, we were able to determine the presence or absence of diastolic dysfunction in 97% (*n* = 240) and were able to determine the grade of diastolic dysfunction in 85% (*n* = 210) (Figure 1).

We identified 70.9% (*n* = 88) in the presternotomy assessment and 75% (*n* = 93) in the poststernal closure assessment as having diastolic dysfunction. We identified normal diastolic function (DFN) at both stages in 20.1% (*n* = 25). Data were indeterminate for the presence or absence of diastolic dysfunction (IDDD) in 3.2% (*n* = 4) at each time point.

Overall, 58.8% of the dataset exhibited no change in diastolic function status (normal or abnormal) between measurements, 20.9% changed by one grade, (improvement in 8.8%, and deterioration in 12.1%), and in 20%, change was considered indeterminable. Changes in diastolic function between the two measurement stages are outlined (Appendix D).

**Figure 2:**
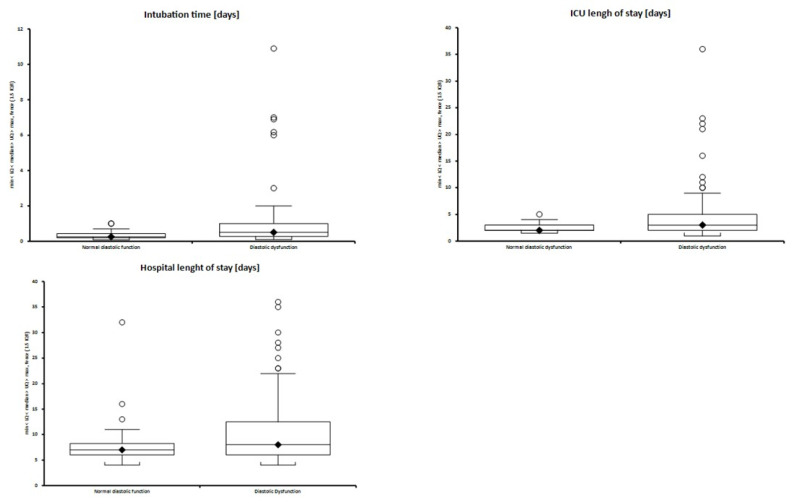
Median and interquartile range of intubation duration, intensive care length of stay, and hospital length of stay in patients with any grade of diastolic dysfunction compared to normal diastolic function. Black square-median value, circle-outliers.

Presence of diastolic dysfunction of any grade or at either time point was associated with greater composite morbidity burden. This included greater cardiac postoperative morbidity scores (CPOMS) on postoperative D5 (3.04 ± 2.31 vs. 4.73 ± 7.18; *p =* 0.009) and D8 (2.83 ± 7.76 vs. 5.23 ± 8.62; *p* = 0.009), and overall CPOMS that were higher by 1.24 (*p* = 0.01) unadjusted, and by 1.14 (SE 0.46, *p* = 0.01) after adjustment for age, bypass time and elective vs. nonelective surgery in patients with diastolic dysfunction versus normal function. In addition, longer durations of intubation (0.25 (0.2–0.42) vs. 0.5 (0.27–1) days; *p* = 0.001, adjusted coefficient 0.52 (SE 0.12, *p* = 0.02)), ICU length of stay (2 (2–3) vs. 3 (2–5) days; *p* = 0.019, adjusted b (SE) = 1.02 (0.46) *p* = 0.025) and new postoperative atrial fibrillation (*p* = 0.043, OR (95% CI) = 3.58 (1.06–12.13) after adjustment for age, bypass time and elective surgery) were observed in those with diastolic dysfunction (Figure 2 and Table 2).

**Figure 3:**
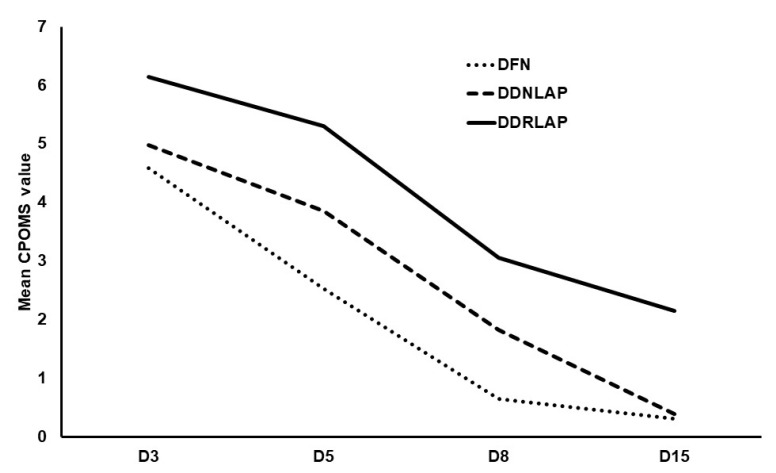
Diastolic category and Cardiac Postoperative Morbidity Score (CPOMS). Normal diastolic function (DFN); diastolic dysfunction with normal left atrial pressure (DDNLAP); diastolic dysfunction with raised left atrial pressure (DDRLAP).

Patients with normal diastolic function and diastolic dysfunction with normal and raised left atrial pressure were compared. With reference to presternotomy measurements, increased severity of diastolic dysfunction, i.e., diastolic dysfunction with raised left atrial pressure (DD2 or DD3 or DDRLAP), versus DD1, was associated with greater morbidity burden for all morbidity outcomes except new postoperative AF (Table 2). CPOMS increased by 1.05 for each category increase in diastolic dysfunction, (*p* < 0.001) (Figure 3).

A multivariable regression model was fitted to identify independent predictors of prolonged length of stay. Variables found to be independently associated with hospital length of stay were age and cardiopulmonary bypass time (Table 3). After adjustment for these variables, diastolic dysfunction was associated with a nonsignificant 15.4% increase in hospital length of stay, (95% CI, −8.2–2.01) *p* = 0.217. By using the length of stay as a categorical variable (>11 days versus <11 days) and fitting a logistic regression model, diastolic dysfunction was not identified as an independent predictor of prolonged length of stay: OR 2.29 (0.69–7.59) *p* = 0.175. No statistically significant independent predictors of prolonged length of stay (>11 days) were identified in the dataset.

## 4. Discussion

The use of the 13-domain CPOMS at defined time points (D3, 5, 8, 15) postoperatively enabled functional indices of morbidity burden to be quantified and compared with patients without diastolic dysfunction for the first time. This study demonstrated that any grade of diastolic dysfunction was associated with greater all-cause morbidity, compared with patients with normal diastolic function. This included CPOMS on days 5 and 8, as well as the length of ICU stay, duration of mechanical ventilation, MACCE, and postoperative atrial fibrillation. In addition, at each measured time point, the CPOMS in patients with diastolic dysfunction was higher by more than one point, amounting to one extra domain or organ system of morbidity, compared with patients with normal diastolic function. CPOMS was also observed to increase by approximately one point with each stepwise increase from normal diastolic function, to diastolic dysfunction with normal left atrial pressure, to diastolic dysfunction with raised left atrial pressure in both presternotomy and poststernal closure groups. The spread of morbidity seemed to suggest a preponderance of renal, cardiovascular, and infection morbidity domains with this pattern occurring in the early and later operative assessment times. Overall, the results confirm that the presence and severity of diastolic dysfunction are associated with major morbidity.

### 4.1. Hospital Length of Stay

Almashrafi et al. recently observed that hospital length of stay after cardiac surgery increases monotonically with the number of postoperative complications [18]. It could be expected that an increase in all-cause morbidity would be associated with a statistically significant increase in length of stay and that the presence of diastolic dysfunction may independently predict the prolonged length of stay (>11 days). Although we found worsening diastolic dysfunction was associated with increased hospital length of stay, it did not independently predict the prolonged length of stay. It is difficult to separate the presence of diastolic dysfunction from some of the other factors that influence the length of stay, such as age, frailty, and severity of disease, and it is notable that none of the measured parameters independently predicted “prolonged” stay. We chose 11 days to offer consistency with other published data; however, the expected length of stay of an individual is procedure specific and also influenced by age and presence of comorbid diseases. Finally, the effect of diastolic dysfunction on prolonged length of stay may be too subtle to be identified in this cohort of patients with the statistical power available.

Although diastolic dysfunction was not identified as an independent predictor of prolonged length of stay, a clear morbidity signal was observed in this perioperative patient population, and we believe that diastolic dysfunction should continue to be studied in this context and will benefit from ongoing analysis in larger-scale studies.

### 4.2. Approaches to the Measurement of Diastolic Function

The assessment of diastolic dysfunction in the perioperative environment is influenced by loading conditions, fluctuating hemodynamics, and mechanical ventilation [19,20,21,22,23,24], and the complexity of algorithms for grading mean guidelines are not necessarily applicable in the perioperative setting [16]. Simplified processes for the assessment of diastolic dysfunction in cardiac surgical patients, independent of loading conditions, have been developed. Swaminathan et al. proposed an algorithm using annular tissue Doppler and transmitral E velocities (e’ and E/e’) to diagnose and grade diastolic dysfunction in 900 CABG patients, which was predictive of longer-term MACCE [5]. The utility of this simplified algorithm was applied to ICU patients with septic shock and found to classify more patients than the traditional algorithm [25].

In 2016, the ASE/EACVI guidelines were updated and simplified, with emphasis given to “2D imaging”, “clinical context”, and four diastolic indices: tricuspid regurgitant jet velocity, indexed left atrial volume, annular e’ velocity, and E/e’. Clinically, diastolic dysfunction is considered present when left ventricular systolic dysfunction (LVEF < 50%) or “myocardial disease” exists. Once diastolic dysfunction is diagnosed, the guidelines place emphasis on a distinction being made between diastolic dysfunction with normal or raised left atrial pressure. Clearly, such clinical context may be observed in many patients presenting for cardiac surgery; the guidelines reference a nonapplicability to the perioperative environment but with no specific statement not to use them. A recent critical care environment meta-analysis applied these guidelines in patients with septic shock and found the identification of diastolic dysfunction was strongly associated with mortality [26]. A patient with septic shock in a critical care environment similarly presents with complex if not fluctuating hemodynamics [27]. We demonstrated that these updated and simplified guidelines can be applied to the perioperative cardiac surgical environment.

### 4.3. Feasibility of Measuring Diastolic Function in a Cardiac Surgical Population

The updated 2016 ASE/EACVI approach enabled us to straightforwardly classify 97.6% of our mixed cardiac surgical dataset, including those patients with mitral valve disease (*n* = 15), with only a very small number of patients ultimately deemed unclassifiable (IDDD). The additional emphasis on clinical context and 2D data, together with the assessment of diastolic indices, has enabled diagnosis and classification in cases where ambiguity would otherwise exist. Importantly, we found the main outcomes of classification: normal diastolic function, diastolic dysfunction with normal left atrial pressure, and diastolic dysfunction with raised left atrial pressure are associated with multisystem morbidity outcomes. We identified the presence of perioperative diastolic dysfunction in 77.4% of our dataset, which is in line with that previously described in a CABG population [2,28]. This is not unexpected given the substantial proportion of patients with coronary arterial disease and indeed myocardial disease in the study. Using the updated guidance will necessarily attribute diastolic dysfunction to most patients, as they will likely have a myocardial disease, and this may well be one of the factors leading toward indication for surgery.

It is important to acknowledge that the two groups—normal diastolic function and diastolic dysfunction—contain different patient characteristics. Age was significantly higher in patients with diastolic dysfunction (*p* = 0.02), although this is not surprising, as diastolic dysfunction is more prevalent with advancing age [2]. It is likely that age reflects the “clinical context” component to diagnosis, as an impaired systolic function or myocardial disease are seen more frequently in older patients. Age alone was not a determining factor for diastolic dysfunction, however, nor was it a feature in algorithm A or B of the 2016 guidelines. Similarly, the presence of hypertension, RWMAs, NHYA > 2 scores, and impaired LV systolic function were significantly higher in patients with diastolic dysfunction, as these are all features strongly associated with abnormal diastology. No patients with impaired ejection fraction were in the normal diastolic function group, as impaired ejection fraction, by definition, allocates the patient to algorithm B of the ASE guidelines (Appendix C). These factors demonstrate that it is very difficult to separate diastolic dysfunction from the clinical pathologies that predispose to it.

### 4.4. Limitations

We acknowledge these findings need to be viewed in the context of limitations. Firstly, our initial primary outcome measure was to assess the effect of enoximone on change in diastolic function and provide a background for future studies of its effect. As our primary outcome measure was undeliverable, our secondary outcome was subsequently powered for this study. We performed a prospective observational study in a nonhomogeneous population of perioperative cardiac surgical patients that included assessment of diastolic function in elective and nonelective or urgent cases, isolated CABGs, isolated valves (including mitral where mitral stenosis and mitral regurgitation may require different echocardiographic diagnostic algorithm; Appendix C) and mixed CABG and valve procedures. We recognize that in nonelective cases, the systolic and diastolic functions of the heart could differ from standard conditions and that in the post cardiopulmonary bypass phase acute diastolic dysfunction is different from chronic diastolic dysfunction. We saw merit in assessing diastolic function across a generic cardiac surgical population, as diastolic dysfunction does not lie in one specific group, although its generic nature may itself have incurred biases. Secondly, although the echocardiographic measurements were taken during the period of hemodynamic stability, we cannot exclude the impact of preload and fluid management on the diastolic function. We did not collect echocardiographic data on fluid responsiveness, such as changes in the stroke volume by velocity-time integral, which could have provided more robust data regarding the volume status. Thirdly, just over one-third of patients (*n* = 48) received a phosphodiesterase inhibitor “on pump”, which may have influenced diastolic measurements acquired after sternal closure. However, we found any grade of diastolic dysfunction measured at either stage pre- or poststernotomy to have a similar effect on the outcome. Change in diastolic function (improved or worse) was observed in 20% of patients receiving phosphodiesterase inhibitors. As the study was not intended as an interventional study, the impact on the perioperative management of diastolic dysfunction needs to be evaluated in future studies. Finally, with regard to the updated ASE/EACVI guidelines on the assessment of diastolic function, we acknowledge these are validated in an outpatient rather than perioperative population and have justified applying them in the discussion above. However, we could not identify a definition of “myocardial disease” within the guidelines, and therefore, an assumption was made about this definition, which may have introduced bias.

## 5. Conclusions

In summary, we were able to demonstrate the feasibility to apply the updated ASE/EACVI guidelines and that grading of diastolic dysfunction could be applied to nearly every patient. Although identification of intraoperative diastolic dysfunction appears to be associated with outcome, its role in perioperative settings remains unknown. Further studies are required to determine whether manipulation and improvement of diastolic dysfunction in the intraoperative phase can result in improved postoperative recovery.

## Figures and Tables

**Table 1:** Distribution of baseline characteristics according to diastolic function. Expressed as % (number), and median (range).

	Normal Diastolic Function (*n* = 25)	Diastolic Dysfunction (*n* = 96)	*p* Value
**Patient characteristics**			
Male	92% (23)	78.1% (75)	*p* = 0.16
BMI	27.8 (23.1–31.2)	28.7 (26–32.4)	*p* = 0.12
Age	**61.8 (54.0–70.6)**	**68.8 (61.3–76.9)**	***p* = 0.021**
Diabetes	28.0% (7)	33.3% (32)	*p* = 0.81
Hyperlipidemia	**52.0% (13)**	**78.1% (75)**	***p* = 0.01**
Hypertension	**32.0% (8)**	**80.2% (77)**	***p* < 0.001**
NYHA	**36.4% (8)**	**77.2% (71)**	***p* < 0.001**
RWMAs	**6.3% (1)**	**55.4% (41)**	***p* < 0.001**
EF < 50%	**0 (0)**	**30.2% (29)**	***p* = 0.001**
**Medication**			
Beta blockers	58.3% (14)	63.5% (61)	*p* = 0.65
Ca channel blockers	20.0% (5)	30.5% (29)	*p* = 0.45
Nitrates	24.0% (6)	32.3% (31)	*p* = 0.48
Statins	72.0% (18)	78.1% (75)	*p* = 0.60
ACE inhibitors	52.0% (13)	70.8% (68)	*p* = 0.10
Diuretics	8.0% (2)	25.0% (24)	*p* = 0.10
**Surgery**			
CPB time	90 (80–104)	88.5 (74–108)	*p* = 0.77
X-clamp time	65 (39–80)	65 (50–83)	*p* = 0.66
Electives	64.0% (16)	65.6% (63)	*p* = 1.00

In bold *p* < 0.05.

**Table 2:** Diastolic function at any time and outcome.

	*n*	Intubation	New AF	HLOS	ICU LOS	MACCE	D3	D5	D8	D15
Normal diastolic function	25	0.25 (0.2–0.42)	3 (12.0%)	7 (6–8)	2 (2–3)	0 (0%)	5 (3–6)	2 (1–5)	0 (0–0)	0 (0–0)
Diastolic dysfunction (any grade)	96	0.5 (0.27–1)	36 (38.3%)	8 (6–12.5)	3 (2–5)	5 (5.2%)	6 (3–8)	4.5 (2–6)	0 (0–4)	0 (0–0)
		***p* = 0.001**	***p* = 0.016**	*p* = 0.055	***p* = 0.019**	*p* = 0.582	*p* = 0.12	***p* = 0.009**	***p* = 0.009**	*p* = 0.14
Indeterminate diastolic dysfunction	3	(2.41%)								
Atrial fibrillation OR (95% CI) = 4.50 (1.22–25.17) *p* = 0.018										

Atrial fibrillation (AF); hospital length of stay (HLOS); intensive care unit length of stay (ICULOS); 30-day major adverse cardiovascular and cerebral events (MAACE); day 3 (D3); day 5 (D5); day 8 (D8); day 15 (D15). In bold *p* < 0.05.

**Table 3:** Multivariate model for length of stay. In bold *p* < 0.05.

	% Difference for Length of Stay (95% CI) (Preadjustment)	*p* Value	Odds Ratio for Prolonged Length of Stay >11 d (95% CI)	*p* Value
Age (per year)	**1.2% (0.4, 1.9)**	**0.003**	1.04 (1.00–1.09)	0.03
Bypass time (per min)	**−25.2% (−38.8, −8.5)**	**0.005**	0.55 (0.22–1.40)	0.21
Non-elective vs. Elective	**24.5% (1.7, 52.5)**	**0.034**	1.10 (0.42–2.90)	0.85
Diastolic dysfunction vs. normal diastolic function	12.1% (−10.4, 40.2)	0.32	2.02 (0.61–6.66)	0.25

## Appendix A

**Table A1:** Cardiac Postoperative Morbidity Score Criteria.

	Cardiac Postoperative Morbidity Score				(13 Domains: Maximum 1 Point for Each Domain)
Morbidity Type	CPOMS Criteria				
Pulmonary	Presence of one or more of the following:				
	New requirement for oxygen or respiratory support (inc. nebulizers/chest physiotherapy on or after D5)				
	Pleural effusion requiring drainage				
Infectious	Presence of one or more of the following:				
	Currently on antibiotics				
	Has had temperature > 38 °C in last 24 h				
	Has had a white count or CRP requiring in-hospital review or treatment				
Renal	Presence of one or more of the following:				
	Decreased urine output requiring intervention (inc. IV furosemide)				
	Increased serum creatinine (>30% from preoperative level)				
	Urinary catheter in situ				
	New urinary incontinence				
	Serum potassium abnormalities requiring treatment				
Gastrointestinal	Presence of one or more of the following:				
	Unable to tolerate an enteral diet for any reason inc nausea, vomiting, abdominal distension				
	Presence of nasogastric tube				
	Diagnosis of gastrointestinal bleeding				
Cardiovascular	Presence of one or more of the following:				
	Use of inotropic therapy for any cardiovascular cause				
	Presence of pacing wires on or after D5 +/− requiring temporary/new permanent pacing				
	Diagnostic tests/Rx in last 24 h for:			(1) New MI/ischemia	
				(2) Hypotension	
				(3) Arrhythmia (req fluids, pharmacotherapy/omission of pharmacotherapy)	
				(4) Cardiogenic pulmonary oedema	
				(5) Hypertension (requiring pharmacotherapy or omission of pharmacotherapy)	
				(6) Thrombotic event (requiring anticoagulation)	
Neurological	Presence of one or more of the following:				
	New neurological deficit				
	(Confusion, delirium, coma, incoordination, drowsy, poor swallow, diplopia, sedation, fluctuating consciousness)				
Haematological	Presence of one or more of the following:				
	Untherapeutic INR requiring pharmacological Rx or omission of pharmacological Rx				
	Requirment in last 24 h for packed red cells, platelets, fresh frozen plasma, cryoprecipitate				
Wound	Presence of one or more of the following:				
	Wound dehiscence requiring surgical exploration/drainage of pus +/− isolation of organisms				
	Presence of chest drains				
	Wound pain significant enough to require continuing or escalating analgesic intervention				
Pain	Postoperative pain significant enough to require parenteral opioids or escalating analgesia				
Endocrine	New or additional requirements for blood sugar management				
Electrolyte	Electrolyte imbalance requiring oral or IV intervention (Na/Urea/Phosphate) *				
Review	Remaining in hospital for further review, investigation, or additional procedure				
Assisted ambulation	New or escalated post op requirement for mobility assistance (wheelchair, zimmer, crutches, walking stick)				

CPOMS, Cardiac Postoperative Morbidity Score; IV, intravenous; CRP, C-reactive protein; INR, international normalized ratio; MI, myocardial infarction. * Where abnormalities refer to local clinical ranges.

## Appendix B

**Table A2:** Types of Surgeries. Expressed as % (Number).

Type of Surgery	
Isolated CABG	78 (62.9%)
CABG plus valve (AVR/MVR)	17 (13.7%)
Isolated valve	24 (19.3%)
>1 valve	5 (4%)

## Appendix C

**Table A3:** Process of Evaluating Diastolic Function.

Where to Start?	Action	Outcomes
First consider the following questions:	Myocardial disease *	Go to Algorithm B
	Depressed LVEF (<50%)	Go to Algorithm B
	Special circumstances (AF, mitral stenosis or regurgitation, depressed EF)	Go to Special Circumstances
	None of the above	Go to Algorithm A
**Algorithm**	**Indices to Evaluate**	**Possible Outcomes**
**A** (is DD present?)	Septal E’ < 7, Lateral E’ < 10, E/E’ > 14, TR velocity > 2.8, LAVI > 34	Majority positive = diastolic dysfunction (DD), go to Algorithm B
		Majority negative = normal diastolic function (DFN)
		50% positive = indeterminate if diastolic dysfunction (IDDD)
**B** (DD is present, what is the grade?)	E/A, Septal E < 7 or Lateral E < 10, E/E > 14′, TR Velocity > 2.8, LAVI > 34	E/A <0.8 and E <50cm/s = Grade 1 (DD1)
		E/A > 2.0 = Grade 3 (DD3)
**IF** E/A < 0.8 and E > 50 **OR** E/A 0.8 to 2	Assess E/E’ > 14, TR Velocity > 2.8, LAVI > 34	Majority positive = DD Grade 2 (DD2)
		Majority negative = DD Grade 1 (DD1)
		50% positive = DD of indeterminate grade (DDIDG)
**Special Circumstances**	**Indices to Evaluate**	**Possible Outcomes**
Atrial Fibrillation	Go to A or B. If B algorithm used include E/E’ > 11 cutoff, E/Vp ratio > 1.4. IVRT (<65 msec)	As per Algorithm A, but if B used, as per AF modification:
		Majority positive = DDRLAP
		Majority negative = DD1
		50% positive = DDIDG
Mitral Stenosis	In Algorithm B, add IVRT < 60	As per A or B outcomes if sinus rhythm, or as per AF modification if in AF
Mitral Regurgitation	Only use E/E’ if depressed EF, add Ardur-Adur (>30 msec) and IVRT (<60 msec) in Algorithm B	As per A or B outcomes if sinus rhythm, or as per AF modification if in AF
Depressed EF	Go to B. Any missing parameters S/D < 0.8 can be used	As per Algorithm B outcomes

* Myocardial disease definition: Hypertension, NHYA > 2, and RWMAs on preoperative echocardiogram. Diastolic dysfunction (DD); grade 1 diastolic dysfunction (DD1); grade 2 diastolic dysfunction (DD2); grade 3 diastolic dysfunction (DD3); normal diastolic function (DFN); indeterminate if diastolic dysfunction (IDDD); diastolic dysfunction of indeterminate grade (DDIDG); diastolic dysfunction with raised left atrial pressure (DDRLAP); left ventricular ejection fraction (LVEF); septal annular velocity (septal E’); lateral annular velocity (lateral E’); peak E velocity (E); tricuspid regurgitant jet velocity (TR velocity); indexed left atrial volume (LAVI).

## Appendix D

**Table A4:** Diastolic Function Breakdown: Presternotomy and Poststernal Closure.

Presternotomy	n = 124	Poststernal Closure	n = 124
DFN	32	DFN	27
DD1	58	DD1	51
DD2	8	DD2	14
DD3	10	DD3	6
DDRLAP	1	DDRLAP	3
DDIDG	11	DDIDG	19
IDDD	4	IDDD	4

Normal diastolic function (DFN); grade 1 diastolic dysfunction (DD1); grade 2 diastolic dysfunction (DD2); grade 3 diastolic dysfunction (DD3); diastolic dysfunction with raised left atrial pressure (DDRLAP); diastolic dysfunction of indeterminate grade (DDIDG); indeterminate if diastolic dysfunction is present, i.e., indeterminate function (IDDD).
